# Buckling of a growing tissue and the emergence of two-dimensional patterns^☆^

**DOI:** 10.1016/j.mbs.2013.09.008

**Published:** 2013-12

**Authors:** M.R. Nelson, J.R. King, O.E. Jensen

**Affiliations:** a School of Mathematical Sciences, University of Nottingham, University Park, Nottingham NG7 2RD, UK; b School of Mathematics, University of Manchester, Oxford Road, Manchester M13 9PL, UK

**Keywords:** Buckling, Tissue growth, Pattern formation, von Kármán plate

## Abstract

The process of biological growth and the associated generation of residual stress has previously been considered as a driving mechanism for tissue buckling and pattern selection in numerous areas of biology. Here, we develop a two-dimensional thin plate theory to simulate the growth of cultured intestinal epithelial cells on a deformable substrate, with the goal of elucidating how a tissue engineer might best recreate the regular array of invaginations (crypts of Lieberkühn) found in the wall of the mammalian intestine. We extend the standard von Kármán equations to incorporate inhomogeneity in the plate’s mechanical properties and surface stresses applied to the substrate by cell proliferation. We determine numerically the configurations of a homogeneous plate under uniform cell growth, and show how tethering to an underlying elastic foundation can be used to promote higher-order buckled configurations. We then examine the independent effects of localised softening of the substrate and spatial patterning of cellular growth, demonstrating that (within a two-dimensional framework, and contrary to the predictions of one-dimensional models) growth patterning constitutes a more viable mechanism for control of crypt distribution than does material inhomogeneity.

## Highlights

• We model the growth of gut epithelial cells cultured upon a deformable substrate.
• Growth generates buckling instabilities, contributing to crypt formation *in vivo*.
• Variations in mechanical properties have little effect on resulting configurations.
• Configurations are controlled by growth patterns & interactions with strata below.

## 1 Introduction

This article addresses the mechanism by which biological growth may generate a build-up of residual stresses within a tissue, the relief of which drives deformations. This patterning mechanism has been previously linked to a vast array of biological systems, including plant leaves, stems and petals [1–3], skin wrinkling [4], and cortical development [5]. Here, our primary interest lies in the formation of the regular array of test-tube-shaped invaginations found in the interior wall of the human large intestine. These *crypts of Lieberkühn* (see *e.g.* Figure 3 in [6]) are known to form approximately seven days after birth in mice; prior to this the intestinal wall is smooth [7,8]. In contrast, the chick embryo exhibits the formation of ridges that develop a zig-zag pattern before turning into undulations that subsequently form villi [9]. The mechanisms that underlie crypt formation are not understood definitively; candidate mechanisms include mechanical buckling instabilities [9], viscous fingering instabilities [10], and Turing instabilities [11]. Here, we consider a contributing biomechanical process through which the cells in the developing intestine’s epithelial lining proliferate and expand, resulting in the whole layer becoming compressed, and ultimately generating out-of-plane deformations.

The crypts of Lieberkühn are responsible for the maintenance of the healthy epithelium: the base of each crypt houses a population of stem cells which continuously produce new epithelial cells. These epithelial cells migrate up the crypt axis as they differentiate and, on arrival at the crypt opening, ultimately undergo programmed cell death and are released into the intestinal lumen [12]. This constant production, differentiation, migration and loss of epithelial cells results in a full regeneration of the layer every 5–6 days [13]. Malformed or dysfunctional crypts are commonly linked to the onset of intestinal cancer [14–16]. A thorough understanding of the mechanisms underlying crypt formation might therefore be useful in developing new intestinal cancer therapies, for example. This study is motivated by the eventual target of using stem cell and tissue engineering techniques for the successful manufacture of intestinal epithelia *in vitro*, a key question being whether tissue engineers must provide a framework for the crypt structure explicitly, or whether they can instead manipulate the underlying biological processes such that, given the correct environment, a cell population would assemble into crypts spontaneously. Recent experimental studies have identified the latter option as a strong possibility. Examples include the study of Viney et al. [17], in which collagen was used to support the co-culture of epithelial cells and fibroblasts for the culture and study of intestinal nematodes, and the study of Sato et al. [18], in which Matrigel was used to demonstrate the ability of isolated crypt stem cells to generate an individual crypt. Spence et al. [19] demonstrated that, via a series of growth factor manipulations that mimic embryonic intestinal development, a population of human pluripotent stem cells can be directed to differentiate into functional epithelial tissue.

Previous models of the colorectal crypt have often used a cell-based description of the epithelium to study the division, differentiation and migration of stem cell progeny [16,20–22]. Lattice-free models [23–25] have facilitated realistic descriptions of cell division, migration and deformation, representing cells by points at their centres or vertices connected by elastic springs that capture cell–cell interactions. Stochastic [26] and deterministic [27] models for the out-of-plane deformations induced by in-plane stresses have elucidated the effects of *e.g.* hyper-proliferation, crypt budding and fission in the onset of colorectal cancer. Recent models have also investigated the role of tissue curvature in driving cell differentiation [28] and regulating tissue growth rate [29].

Via an experiment in which intestinal epithelial cells were cultured upon a deformable substrate, Nelson et al. [30] validated the hypothesis that cellular proliferation and expansion against fixed boundaries can result in sufficient in-plane compression to generate out-of-plane deformations of intestinal epithelia *in vitro*. Two parallel biomechanical models, each a one-dimensional (1D) representation of a 2D system, were used to demonstrate that buckling thresholds and post-buckled configurations were largely unchanged under variation of cell-substrate adhesion properties or under spatial patterning of the cells’ growth rate. The study showed that mode selection can, instead, be controlled by either patterning the material properties of the substrate, or by tethering the substrate to an underlying foundation. However, these predictions have yet to be fully assessed using more realistic 2D models.

Mathematical models of growing biological organisms have been an active area of research since the 1940s [31,32]. While many early papers deployed geometric arguments to track displacements of material points alone (*e.g.* [33,34]), tissue growth models are now commonly embedded into the theory of nonlinear elasticity. Skalak and Rodriguez [35–37] provided a formalism for this process, suggesting a decomposition of the associated deformation gradient tensor as F=AG, where the tensor *G* captures growth effects (mapping an initially unstressed and unloaded body into an enlarged, and possibly incompatible, configuration), and the tensor *A* accounts for the elastic deformations required to satisfy external constraints and correct for any growth-induced incompatibilities. Later refinements of this framework have separately accounted for growth, remodelling and morphogenesis; for details see [32,38,39] and references therein.

One common approach to modelling growth-driven tissue deformation is to describe thin tissue layers such as epithelia using von Kármán’s equations for thin plates [40,41]. The theory is derived from nonlinear elasticity via a series of assumptions upon the magnitude of each of the stress/strain components, primarily that the magnitudes of transverse stresses and strains are at least O(ε) smaller than those of the in-plane components, for aspect ratio $\varepsilon =h^{*}/L^{*}\ll 1$ (for plate thickness $h^{*}$, width $2L^{*}$). See Appendix A or [42] for further details. The resulting theory holds for transverse displacements of $O(h^{*})$; we term this theory ‘weakly nonlinear’. Hannezo et al. [29] deployed von Kármán theory in their model of the intestinal epithelium, presenting a three-layered model in which the epithelium rests upon a basement membrane mounted on an elastic stroma. Through tuning of the mechanical properties of the elastic stroma, and coupling of the epithelial growth rate to membrane curvature, the authors were able to attain buckled configurations which replicated the geometric differences between the large and small intestine (in which villi also protrude into the lumen).

An alternative approach is to regard the tissue layer as a thin, nonlinear shell, and to derive governing equations via a ‘balance of forces’ formulation [43–46]. While the use of nonlinear shell models is advantageous in facilitating study of larger-amplitude deformations, this approach presents additional complication in terms of selecting and justifying the necessary constitutive assumptions, often from a choice of many possibilities [45].

The model of Dervaux & Ben Amar [47] coupled a von Kármán description of an epidermal layer of skin tissue to a linearly elastic basement membrane, assessing the role of localised tissue growth in pattern selection. Comparing their full model to a 1D reduction in which growth is uni-directional, the authors concluded that under uni-directional growth the profile of the growing tissue is determined entirely by the net growth, becoming independent of the local growth field expression. However, in the full 2D problem, stronger energetic constraints on bending and stretching in two directions restrict attainable patterns, allowing patterned growth to play a stronger role in pattern selection. We revisit this claim here to assess the strength of the conclusions of [30], in which 1D models indicated that patterned growth was not sufficient to control crypt distributions.

In this paper, we extend the 1D models of [30] to two spatial dimensions. Motivated by the experimental formulation therein, we address the question of how a tissue engineer might best manipulate the cell environment *in vitro* toward the goal of generating intestinal epithelia which display the required crypt geometry. Considering a typical cell culture substrate as a thin plate, we present an extension to the standard von Kármán equations to incorporate (i) surface stresses induced by proliferating cells upon the substrate’s upper surface, and a supporting foundation below, and (ii) spatial variations in the plate’s mechanical properties. We present the configurations attained by a homogeneous plate buckling under the influence of a uniformly growing cell layer, and show how these configurations are affected by attachment to a supporting elastic foundation, localised softening of the substrate, and localised cellular growth.

## 2 Model

**Fig. 1:**
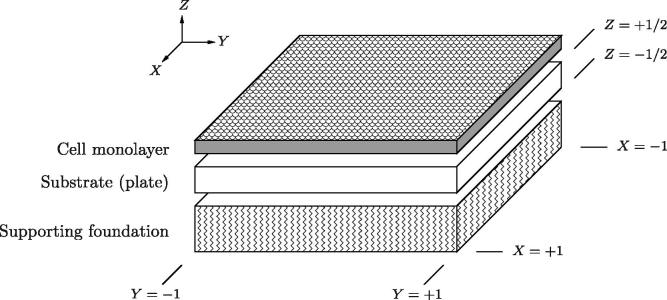
Geometry of the modelled problem in the undeformed configuration. A square cell culture substrate, held between clamps along all four edges, is modelled as a thin plate. A growing cell monolayer rests upon this substrate, which in turn may rest upon a supporting viscous or elastic foundation. Expansion of the cell layer against fixed boundaries may drive deformations of the plate, with the supporting foundation playing a role in shape selection.

We consider a square cell culture substrate clamped between fixed supports along its four edges. Upon the upper surface of the substrate rests a confluent monolayer of epithelial cells (see Fig. 1). Continued proliferation causes the cells to exert sufficient force against the fixed boundaries to deform the substrate and increase the available culture surface area. In general, the substrate may itself be supported by an elastic or viscous foundation.

In its undeformed configuration, the substrate is flat, unstressed and of width $2L^{*}$ and thickness $h^{*}$; we define $\varepsilon =h^{*}/L^{*}\ll 1$. Stars denote dimensional quantities throughout. We denote the substrate’s Young’s modulus by $E^{*}$ and its Poisson ratio by ν∈[0,0.5]. We present the model here in terms of dimensionless variables defined as follows. In-plane and out-of-plane Cartesian coordinates are nondimensionalised against $L^{*}$ and $h^{*}$ respectively; in terms of dimensionless Lagrangian coordinates, the plate is bounded by -1⩽X,Y⩽1 in the plane of the plate, and -1/2⩽Z⩽1/2 in the out-of-plane direction (Fig. 1). Since the substrate is thin, it is conveniently described using von Kármán plate theory. Accordingly, in-plane displacements are assumed to be of $O(\varepsilon h^{*})$, while out-of-plane displacements are assumed to be $O(h^{*})$. Nondimensionalising $E^{*}$ against its maximal value, setting $E^{*}=E_{\max}^{*}E$, we adopt the scalings of [42], assuming that in-plane stresses scale as $\varepsilon^{2}E_{\max}^{*}$, out-of-plane shear stresses scale as $\varepsilon^{3}E_{\max}^{*}$ and out-of-plane normal stresses scale as $\varepsilon^{4}E_{\max}^{*}$. For a full derivation of the model, see Appendix A.

The cell layer and the supporting foundation may exert both in-plane and out-of-plane stresses upon the upper and lower surfaces of the plate respectively. We introduce vectors $\pm f^{\pm }$ to capture these surface stresses (‘+’ referring to the upper surface, ‘−’ the lower). In-plane effects are conveniently described in terms of the quantities

(1) $$F(X\text{,}Y)=f^{+}+f^{-}\text{,}\;\Omega (X\text{,}Y)=f^{+}-f^{-}\text{,}$$

respectively being the total in-plane surface stress applied upon both upper and lower surfaces, and the couple induced by these stresses. As shown in Appendix A, definition of a standard Airy stress function requires the existence of some scalar $\chi \left(X\text{,}Y\right)$ for which F=∇χ; we therefore assume that ∇×F=0.

Normal surface stresses applied upon either surface are captured by the variable N, which incorporates contributions from cell growth above the plate and a support below. We assume that epithelial cell growth involves in-plane expansion with no preferred direction, so that the cell layer is under an isotropic compression T<0. Following [30], we expect this compressive force to be transmitted to the substrate as a normal force proportional to the plate’s curvature, $\nabla^{2}w$. We model the underlying support as a Winkler foundation, a series of elastic springs connecting points on the plate to a series of reference points, providing a restoring force which is linearly proportional to the displacement. Such elastic foundations have previously been used to mediate buckling patterns: examples include [48–50]. We thus consider the following form of N here:

(2) $$N=T\nabla^{2}w+\mathrm{kw}\text{,}$$

where the parameter *k* captures the stiffness of the foundation.

The governing equations for deformation of a spatially inhomogeneous von Kármán plate under the action of the above surface forces are derived in Appendix A. Variables are averaged across the thickness of the plate, and configurations are then described in terms of the plate’s central plane alone. We determine the following equations for the deflection of the plate’s central plane, w(X,Y), and the Airy stress function, Φ(X,Y), which hold to leading order in ε:

(3a) $$\nabla^{2}\left(\frac{1}{E}\nabla^{2}\Phi \right)-\left[\frac{1+\nu }{E}\text{,}\Phi \right]-\nabla^{2}\left(\frac{1-\nu }{E}\chi \right)+\frac{1}{2}\left[w\text{,}w\right]=0\text{,}$$

(3b) $$\nabla^{2}\left(\frac{E}{12\left(1-\nu^{2}\right)}\nabla^{2}w\right)=\left[\frac{E}{12\left(1+\nu \right)}\text{,}w\right]+[w\text{,}\Phi ]+\frac{1}{2}\nabla \cdot \Omega -\nabla \cdot \left(\chi \nabla w\right)+N\text{.}$$

Here [η,Θ] represents the commutator of functions η(X,Y) and Θ(X,Y), given by

(4) $$[\eta \text{,}\Theta ]\equiv \frac{\partial^{2}\eta }{\partial X^{2}}\frac{\partial^{2}\Theta }{\partial Y^{2}}+\frac{\partial^{2}\Theta }{\partial X^{2}}\frac{\partial^{2}\eta }{\partial Y^{2}}-2\frac{\partial^{2}\eta }{\partial X\partial Y}\frac{\partial^{2}\Theta }{\partial X\partial Y}\text{.}$$

We solve (3) subject to ‘clamped’ boundary conditions on all four edges, given by

(5a) $$w=\frac{\partial w}{\partial X}=\Phi =\frac{\partial \Phi }{\partial X}=0\;\mathrm{on}\;X=\pm 1\text{,}$$

(5b) $$w=\frac{\partial w}{\partial Y}=\Phi =\frac{\partial \Phi }{\partial Y}=0\;\mathrm{on}\;Y=\pm 1\text{.}$$

For justification of these boundary conditions, and discussion of alternatives, the reader is directed to [51].

In the sections below, configurations are examined under various choices of the model’s six inputs: E,ν,χ,Ω,T and *k* (all of which can in general vary spatially). We restrict attention to the onset of buckling to address the question of which of the model’s components is most effective in promoting deviations from the flat state. We thus examine appropriate linearisations of (3)–(5). Model outputs *w* and Φ are computed via numerical solution of (3)–(5) via spectral methods in Matlab as described in [52].

In Sections 3.1 and 3.2, we consider the case in which there is no adhesion between the cell layer and the substrate (χ=0,Ω=0). In this case, the cell layer is under a uniform compression (*T* constant) and configurations are independent of spatial variations in cell growth. Firstly, in Section 3.1, we consider the buckling instabilities of a homogeneous plate (E≡1) in the absence of a supporting foundation (k=0). In this limit, (3) reduces to the standard von Kármán equations. In Section 3.1.1, we demonstrate how the addition of an underlying foundation (k≠0) can be used to promote certain higher-order modes. In Section 3.2, we assess the extent to which non-uniform material softening can influence deformations. With k=0, we choose *E* to vary spatially such that specified regions of the substrate exhibit a reduced resistance to deformation.

Then, in Section 3.3, we examine the manner in which patterned cellular growth affects the buckling patterns of a homogeneous plate (for which E≡1). Since transmission of localised growth forces to the substrate requires some adhesion between the layers, *χ* and Ω are non-zero in this case. As described in Appendix B, we modify the plate equations (3a) and (3b) to incorporate growth via a multiplicative decomposition of the deformation gradient tensor into elastic and growth-induced components, as formalised by Skalak & Rodriguez [35–37]. We construct a simple two-layered variant of our model, in which both the cell layer and the substrate are modelled as von Kármán plates. Both layers are considered homogeneous, and deflections are driven by growth of the cell layer alone. We distinguish quantities relating to the cell-layer and the substrate through ‘*c*’ and ‘*s*’ subscripts respectively. We neglect the supporting foundation, setting $f_{s}^{-}=0$, so that $\Omega_{s}=\nabla \chi_{s}$. In-plane surface forces arise solely due to the friction between the two layers; below, we write $\chi_{s}=-\chi_{c}\equiv \chi$ for concision. The layers both undergo deflection *w*; stresses in the cell layer and substrate are denoted $\Phi_{c}$ and $\Phi_{s}$ respectively. We consider in-plane isotropic growth of the cell layer, which we include through a new function g(X,Y), the resulting system of equations being

(6a) $$\nabla^{4}\Phi_{c}+\left(1-\nu_{c}\right)\nabla^{2}\chi +\frac{1}{2}[w\text{,}w]+\left(1-\nu_{c}\right)\nabla^{2}g=0\text{,}$$

(6b) $$\nabla^{4}\Phi_{s}-\left(1-\nu_{s}\right)\nabla^{2}\chi +\frac{1}{2}[w\text{,}w]=0\text{,}$$

(6c) $$\frac{1}{12\left(1-\nu_{s}^{2}\right)}\nabla^{4}w=[w\text{,}\Phi_{s}]+\frac{1}{2}\nabla^{2}\chi -\nabla \cdot \left(\chi \nabla w\right)+N\text{.}$$

In (6a), g(X,Y) describes the distribution of stresses associated with the flat configuration; as the cells grow, *g* increases and the layer must deform in order to relieve the induced in-plane compression. Eq. (6c) describes deflections of the central plane of the substrate, driven by corresponding in-plane stresses ($\Phi_{s}$) and the action of the growing cell layer. In-plane stresses in the cell layer ($\Phi_{c}$) are transferred to the substrate via surface stresses *χ* and N; as cellular stresses accumulate, *χ* and N drive substrate deformation. In Section 3.3, we linearise the system given by (5) and (6) to examine the onset of buckling driven by patterned cell growth.

## 3 Results

### 3.1 Homogeneous plate, uniform cellular growth

We begin our analysis with the simplest two-dimensional case: that of a homogeneous plate (E≡1,ν constant), upon which rests a cell monolayer which exerts no drag upon the plate’s surface (χ=0,Ω=0). Buckling instabilities are driven by the normal force exerted upon the plate as the cell layer expands against fixed boundaries. Since the cells are free to slide against the plate, configurations are independent of the patterning of cellular growth; only the net growth matters. To determine the degree of cellular compression required to generate a buckling instability, we linearise (3), setting $w=\zeta \overset{¯}{w}$ and $\Phi =\zeta^{2}\overset{¯}{\Phi }$ for some 0<ζ≪1. Under the above assumptions, the leading-order terms of Eq. (3) give (omitting bars)

(7) $$\nabla^{4}w+\xi \nabla^{2}w-\eta w=0\text{,}$$

where $\xi =-12T\left(1-\nu^{2}\right)>0$ and $\eta =12k\left(1-\nu^{2}\right)>0$. Analytical solution of (7) subject to (5) is not possible in general, since the clamped boundary conditions do not permit the existence of a separable solution [53].

We consider first the case η=0, for which no supporting foundation is present; we return to the case η≠0 in Section 3.1.1 below. As described by Hoyle [54], we may categorise solutions to (7) in terms of irreducible representations of the dihedral group $D_{4}$, which describes symmetries of a square. There exist five categories of solution, which we label $R_{1}\text{,}\ldots \text{,}R_{5}$, with properties as follows. $R_{1}$ encompasses solutions which are invariant under both reflection about X=0 and rotation by π/2 radians about the origin. $R_{2}$ corresponds to solutions which are negated under this rotation but preserved under this reflection. Solutions which are negated under this reflection and invariant under this rotation belong to $R_{3}$, while $R_{4}$ solutions are negated under both actions. $R_{1}$ and $R_{2}$ solutions are even in both *X* and *Y*, while $R_{3}$ and $R_{4}$ are odd in both *X* and *Y*. The final category, $R_{5}$ incorporates all remaining solutions. Solutions in the $R_{5}$ classification correspond to repeated eigenvalues of (7); taking linear combinations of the associated modes yields a variety of configurations. Below, we show solutions in two sub-categories, $R_{5a}$ and $R_{5b}$, which each exhibit symmetry about one of the square diagonals. Taking linear combinations of $R_{5a}$ and $R_{5b}$ can generate configurations with horizontal or vertical symmetry (omitted below). We introduce the terminology “eigenmode $R_{i}^{\left(j\right)}$” to describe the $j^{\mathrm{th}}$ eigenmode in the $R_{i}$ family (i∈[1,5],j∈[1,∞)); $R_{i}^{\left(1\right)}$ refers to the mode corresponding to the smallest eigenvalue in family $R_{i}$, for example.

**Fig. 2:**
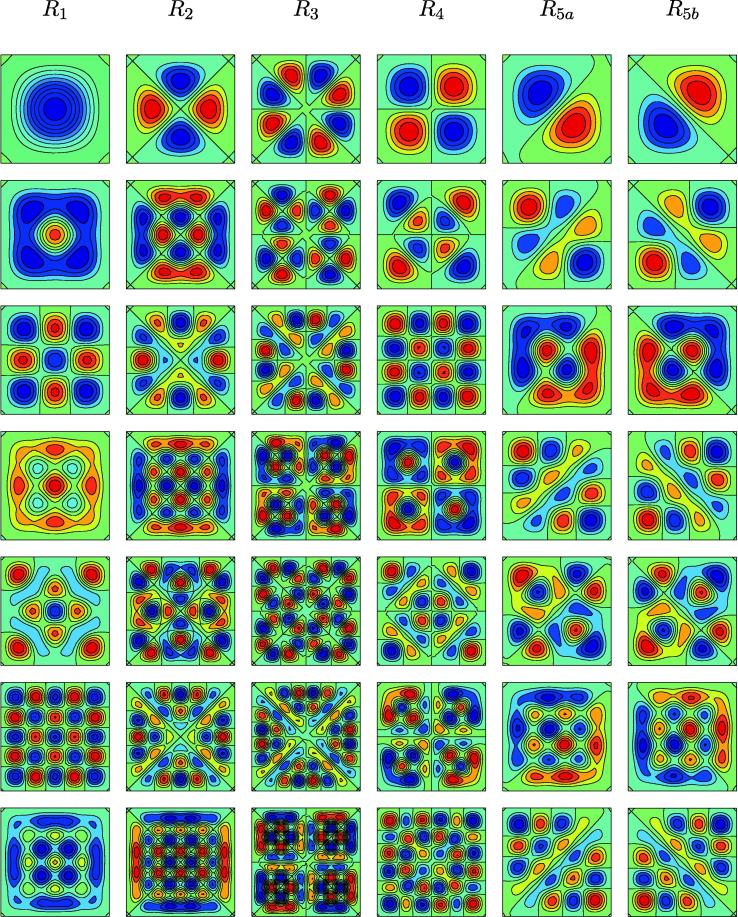
The first seven eigenmodes of (5) and (7) with η=0 for each representation of $D_{4}$, corresponding to the eigenvalues given in Table 1. Solutions are categorised according to their symmetries under reflection about X=0 and rotation about π/2 radians. Dark blue (*resp.* red) colouring represents unit downward (*resp.* upward) buckling. (For interpretation of the references to color in this figure legend, the reader is referred to the web version of this article.)

Table 1 gives the first seven eigenvalues, ξ, for eigenmodes classified under each of the five representations of $D_{4}$ described above. These data recover the equivalent results of [53]. The eigenmodes corresponding to the eigenvalues listed in Table 1 are illustrated in Fig. 2. As the onset of cellular growth generates a compression in the cell layer, a normal force is transmitted to the substrate, inducing buckling instabilities for values of *T* which correspond to eigenvalues of (7). The configuration attained for the least force is mode $R_{1}^{\left(1\right)}$, which exhibits a single extremum in the centre of the domain. For an incompressible substrate (ν=0.5), this instability occurs for T=-1.45. For greater degrees of compression (*i.e.* more prolonged cellular growth) we see higher modes; the next mode to appear is of the $R_{5}$ family. Solutions which correspond to $R_{5}$ arise for eigenvalues of multiplicity 2, enabling us to select two linearly independent eigenmodes to treat as basis functions, the linear combination of which (under normalisation) gives rise to a one-parameter family of $R_{5}$ modes for each eigenvalue pair. For values of *T* of large magnitude the system admits highly wrinkled configurations. Fig. 7(a) below further illustrates the shapes of the $R_{1}^{\left(1\right)}$ and $R_{1}^{\left(2\right)}$ configurations, showing cross-sections of these profiles in the line Y=X.

#### 3.1.1 Mode selection via a Winkler foundation

We now illustrate the manner in which mode selection can be mediated by a supporting foundation attached to the underside of the plate. Such a tethering may be representative of the sub-epithelial mucosa *in vivo*, or of a supporting scaffold used for cell culture *in vitro*. In this case, deformations are governed by (7) with η≠0, subject to the boundary conditions (5).

**Fig. 3:**
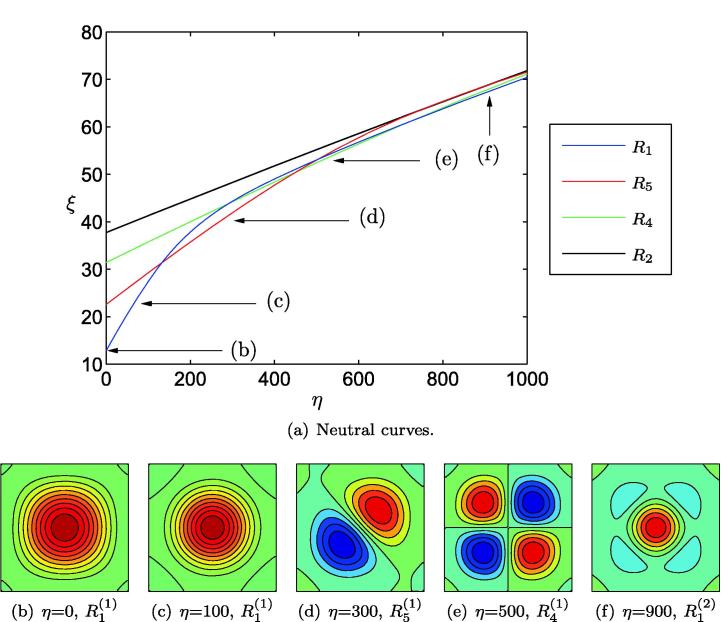
Configurations selected by a homogeneous substrate attached to a Winkler foundation. In (a) we show the first four neutral curves, illustrating the cellular compression, $\xi =-12T\left(1-\nu^{2}\right)$, required to buckle the substrate as a function of foundation stiffness, *η*. In (b)–(f) we illustrate the eigenmode corresponding to the lowest value of *T* for various *η*, as indicated in (a). Dark blue (*resp.* red) colouring represents unit downward (*resp.* upward) buckling. (For interpretation of the references to color in this figure legend, the reader is referred to the web version of this article.)

Fig. 3 illustrates the eigenmodes that are obtained for the least degree of compression in the cell layer as *η* increases. As Fig. 3(a) shows, increasing *η* results in crossings of the neutral curves, resulting in transitions of the first eigenmode from symmetry family $R_{1}$ to $R_{5}$ (at η=134), followed by $R_{4}$ (at η=448) *etc.* Where neutral curves cross, we anticipate the existence of mixed-mode states at finite amplitude, arising through sequences of symmetry-breaking bifurcations. As we travel along any specific neutral curve, the corresponding eigenmode undergoes a series of symmetry-preserving transitions analogous to moving down the corresponding column of Fig. 2. The mode $R_{1}^{\left(1\right)}$ configurations, for example, progress through a sequence of configurations similar to those illustrated in the left-hand column of Fig. 2, the first transition being from the configuration shown in Fig. 3(b) to that of Fig. 3(f) for η=134. This analysis demonstrates that a Winkler foundation can be used to promote higher-order modes, although it is not clear whether it is possible to chose *η* such that an arbitrary desired configuration will become promoted above all others. Within the range of parameters examined here, for example, $R_{2}^{\left(1\right)}$ never becomes the lowest energy configuration. It is unclear as to whether this might occur for higher choices of *η*. Fig. 3 constitutes the two-dimensional analogue of Figure 8 in [30], whose one-dimensional models also demonstrated that the buckling wavelength can be controlled through elastic tethering to an underlying foundation.

### 3.2 Inhomogeneous plate, uniform cellular growth

We now consider a substrate whose mechanical properties vary spatially, assessing the hypothesis that localised tissue softening might be a contributing mechanism in controlling crypt distribution during development. While information regarding matrix stiffness is relatively scarce at the developmental stage, the growth factor TGF-*β* is known to stimulate collagen deposition in tissues, resulting in a stiffening of the extracellular matrix [55]. Since TGF-*β* is expressed in abundance near the top of the crypt, and barely at all near the crypt base [56], the extracellular matrix which surrounds the stem cells in the developed crypt is likely to be less stiff than that elsewhere.

As an illustrative example, we examine buckled configurations for a plate whose Young’s modulus is prescribed according to

(8) $$E(X\text{,}Y)=\frac{1+c-c\exp\left(p{\left(X^{2}-a^{2}\right)}^{2}+p{\left(Y^{2}-a^{2}\right)}^{2}\right)}{1+c-c\exp\left(p{\left(1-a^{2}\right)}^{2}+p{\left(1-a^{2}\right)}^{2}\right)}\text{.}$$

We assume that the Poisson ratio remains uniform. We examine solutions for a=0, for which the substrate has a softened region in the centre, and a=0.5, for which the plate has a distinct softened region in each quadrant. The denominator in (8) is introduced to ensure consistency with (A.13). As *c* is increased from zero, the magnitude of variations in the substrate’s stiffness is increased.

**Fig. 4:**
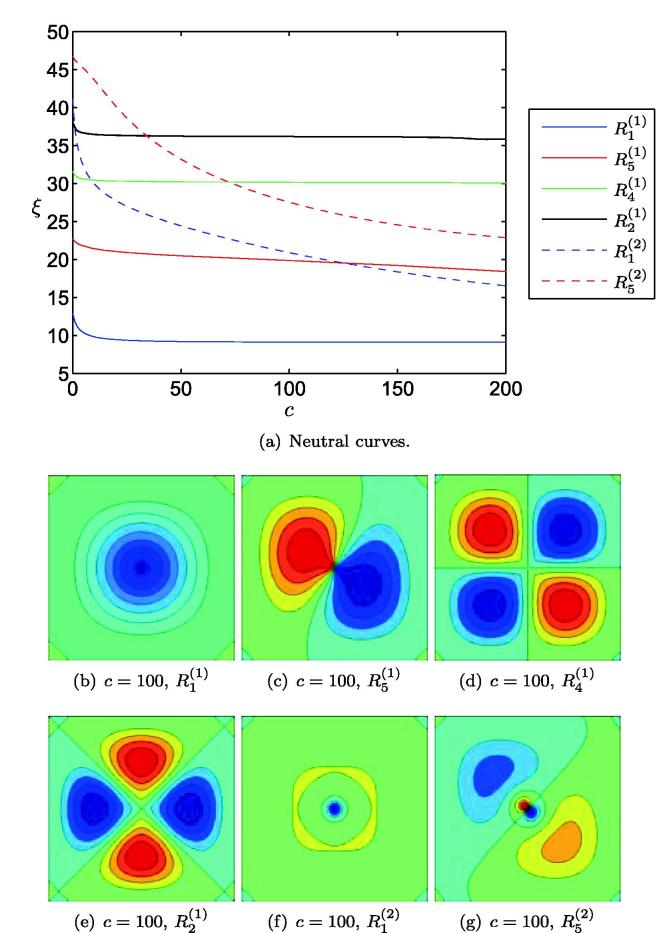
Configurations of a plate with a softened region in the centre, with Young’s modulus given by (8) with a=0,p=-1000, increasing *c* and ν=0.5. In (a) we show the first six neutral curves, illustrating the level of cellular compression ($\xi =-12T(1-\nu^{2})$) required for the plate to buckle, as a function of softening parameter *c*. Panels (b)–(g) show the configurations corresponding to each of these curves for c=100. Dark blue (*resp.* red) colouring represents unit downward (*resp.* upward) buckling. (For interpretation of the references to color in this figure legend, the reader is referred to the web version of this article.)

Fig. 4 illustrates the first six eigenmodes of the system (3), (5) and (8) for a=0 and increasing values of *c*, *i.e.* a plate with an increasingly softened region at its centre. As Fig. 4 shows, softening a region at the centre of the plate permits deformed configurations for a lesser amount of compression in the cell layer. For the parameters investigated, the $R_{1}^{\left(1\right)}$ configuration remains the lowest energy state; however, softening in the centre of the plate does promote some higher-order states, such as $R_{1}^{\left(2\right)}$ and $R_{5}^{\left(2\right)}$, more than others. Softening in the centre of the plate has little effect upon the shape of the $R_{1}^{\left(1\right)}$ configuration; although for larger values of $c\text{,}R_{1}^{\left(2\right)}$ does exhibit a steeper-sided downward protrusion than in the homogeneous case, albeit at the expense of some upward deflection around its perimeter (shown in yellow in Fig. 4(f)).

**Fig. 5:**
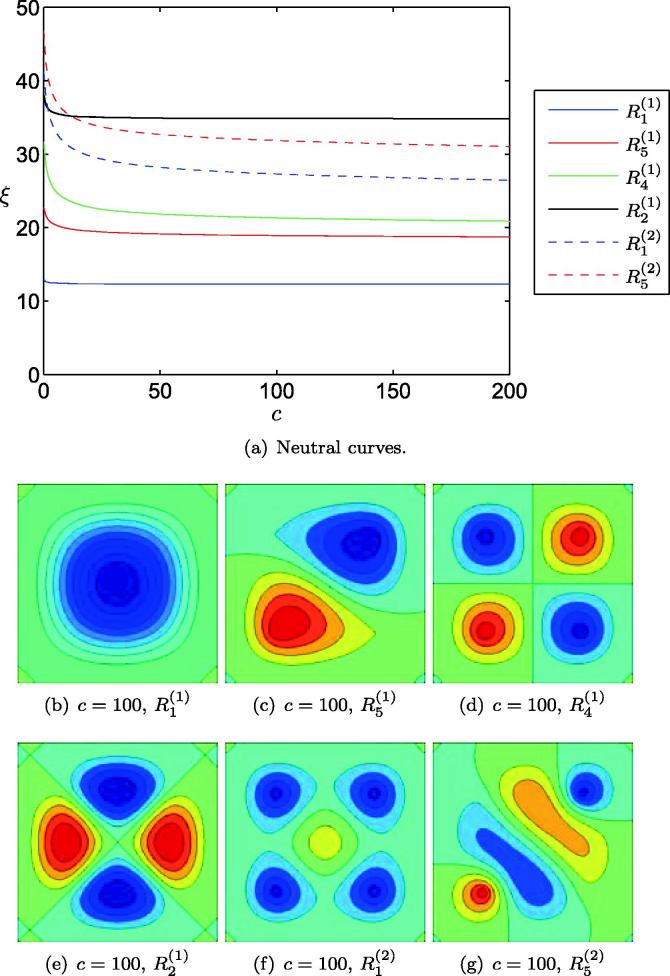
Configurations of a plate with a softened region in each quadrant, with Young’s modulus given by (8) with a=0.5,p=-50, increasing *c* and ν=0.5. In (a) we show the first six neutral curves, illustrating the level of cellular compression ($\xi =-12T(1-\nu^{2})$) required for the plate to buckle, as a function of softening parameter *c*. Panels (b)–(g) illustrate the configurations corresponding to each of these curves for c=100. Dark blue (*resp.* red) colouring represents unit downward (*resp.* upward) buckling. (For interpretation of the references to color in this figure legend, the reader is referred to the web version of this article.)

Similarly, as Fig. 5 shows, softening an area in each quadrant of the plate (via (8) with a=0.5) can promote similar high-order modes. While $R_{1}^{\left(1\right)}$ remains the low energy state for the parameters investigated, $R_{1}^{\left(2\right)}$ and $R_{5}^{\left(2\right)}$ are promoted above $R_{2}^{\left(1\right)}$, for example. The $R_{1}^{\left(2\right)}$ configuration, in particular, displays four distinct downward-pointing protrusions in locations corresponding to the softened areas (Fig. 5(f)). This configuration may be regarded as most akin to multiple crypt shapes within a single plate; however, it appears that significant softening (or interaction with other physical effects, such as an elastic foundation) is required to promote this mode above $R_{1}^{\left(1\right)}$. Fig. 7(b) and (c) below show cross-sections of the $R_{1}^{\left(1\right)}$ and $R_{1}^{\left(2\right)}$ configurations attained in both of the above softening regimes, illustrating the extent to which material softening can yield straighter-sided protrusions.

### 3.3 Homogeneous plate, patterned cellular growth

We now consider the extent to which spatial variations of cellular growth affect deformed configurations, examining solutions to the system given by (5) and (6), with adhesion between the two layers restored.

Linearising (6) about the flat configuration, we assume g,χ and $\Phi_{s}$ are O(w), and that $\Phi_{c}$ and N are $O\left(w^{2}\right)$ (since the additional term in (B.2) keeps cell stresses small). To leading order, we then obtain

(9) $$\frac{1}{12\left(1-\nu_{s}^{2}\right)}\nabla^{4}w=-\frac{1}{2}\nabla^{2}g\text{.}$$

Here, we consider growth which is concentrated at specific locations over the layer, for which we can write g(X,Y) as a sum of $N^{2}$ Gaussians centred at $\left(X\text{,}Y)=(\mu_{i}\text{,}\mu_{j}\right)$ (i,j=1,…,N), *i.e.*

(10) $$g\left(X\text{,}Y\right)=\overset{N}{\underset{i=1}{\sum }}\overset{N}{\underset{j=1}{\sum }}\frac{a}{\sigma \sqrt{2\pi }}\exp\left(-\frac{{\left(X-\mu_{i}\right)}^{2}+{\left(Y-\mu_{j}\right)}^{2}}{2\sigma^{2}}\right)\text{,}$$

in which *σ* controls the Gaussian function’s width, and *a* is an arbitrary scaling factor. Each point $\left(\mu_{i}\text{,}\mu_{j}\right)$ represents one small cluster of proliferating cells. Below, we present results in which $N^{2}$ clusters are arranged in a regular N×N grid with

(11) $$\mu_{i}=-1+\frac{1}{N}\left(2i-1\right)\text{,}\;i=1\text{,}\ldots \text{,}N\text{.}$$

Setting N=1 in (10) and (11) corresponds to a regime in which growth is focused in a single region at the centre of the plate, while setting N=2 corresponds to a distinct region of growth sited in each quadrant of the domain, *etc*. Numerical simulations for other choices of *g* yielded results qualitatively similar to those described below – details are omitted for brevity.

**Fig. 6:**
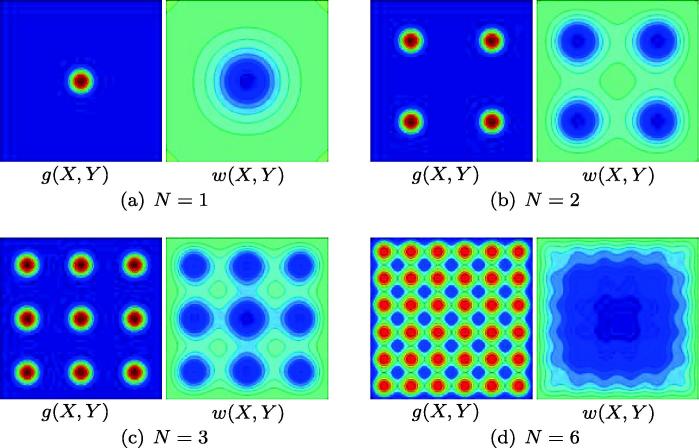
Configurations of a homogeneous plate deformed by a growing cell layer whose growth rate is defined by (10) and (11) with a=10,σ=0.1 and (a) N=1, (b) N=2, (c) N=3, (d) N=6. Dark blue (*resp.* red) colouring represents unit downward (*resp.* upward) buckling. (For interpretation of the references to color in this figure legend, the reader is referred to the web version of this article.)

**Fig. 7:**
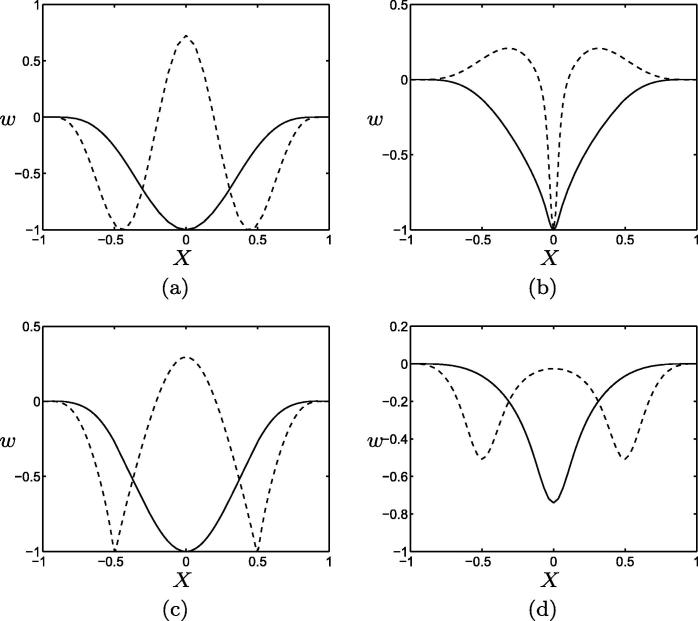
Cross-sections of configurations attained in each regime, in the line Y=X. Plotted in (a–c) are modes $R_{1}^{\left(1\right)}$ (solid lines) and $R_{1}^{\left(2\right)}$ (dashed lines) obtained for (a) a homogeneous plate (*cf.*Fig. 2), (b) a plate softened at its centre (*cf.*Fig. 4 a plate with a softened region in each quadrant (*cf.*Fig. 5(b) and (f)). (d) illustrates particular integrals of (9), showing configurations attained under one/four prescribed patches of growth (*cf.*Fig. 6(a) and (b)). Parameter values are as given in Figs. 2–6.

Fig. 6 shows particular integrals of (9), which perturb the flat configuration according to patterns of localised growth given by (10) and (11) with σ=0.1,a=10 and N=1,2,3 and 6. As Fig. 6(a) shows, restricting growth to a small region in the centre of the plate results in a single downward-pointing protrusion at the same location. In Fig. 6(b) and (c), the plate adopts configurations with four/nine distinct ‘crypts’ having four/nine prescribed patches of localised growth. These results indicate that, in the presence of sufficient adhesion between the cell layer and its substrate, patterning of growth can have a greater degree of control over deformed configurations than the previous one-dimensional models of [30] suggested. For shorter-wavelength growth variations such as that with N=6 (Fig. 6(d)), the local bending energies associated with each region of growth interact, resulting in a configuration reminiscent of a perturbed $R_{1}^{\left(1\right)}$ configuration, rather than the desired regular array of crypts. Cross-sections in the line Y=X of the configurations of Fig. 6(a) and (b) are shown in Fig. 7(d). We remark that more complex solutions may arise when the particular integrals of (9) are combined with solutions to the homogeneous problem, which are similar in shape to the profiles of Fig. 2. In particular, we anticipate a marked increase in the magnitude of the response when growth patterns excite the natural modes.

## 4 Discussion

In this study, we have developed a model to examine the mechanisms of growth-induced buckling in a two-dimensional tissue. The primary motivation for this work lies in understanding the processes that underlie colorectal crypt formation *in vivo*, with a view to replicating intestinal tissue *in vitro*. Specifically, we address the question of how a tissue engineer might best manipulate the tissue culture conditions towards this goal – must a scaffold be structured to provide the crypt geometry *ab initio*, or can crypts form autonomously given an appropriate deformable foundation? Historically, many *in vitro* experiments have taken the former approach, examples including [57,58]; however, recent studies have begun to demonstrate that crypts can form spontaneously in culture given appropriate conditions [17–19].

Motivated by the culture system discussed in [30], the model presented here describes a cell culture substrate via modification of von Kármán’s plate equations to incorporate (i) material inhomogeneities in the plate, and (ii) non-zero surface stresses exerted upon the plate’s upper and lower surfaces by proliferating cells or an underlying foundation. The substrate equations were then coupled to a simple description of a growing cell layer, which rests upon the substrate, and an elastic foundation residing below. This model thus constitutes a natural extension of the 1D models of [30] to two spatial dimensions. Numerical simulations demonstrated the range of eigenmodes that can be obtained via buckling instability in the absence of patterning, and the effects of each patterning mechanism were then elucidated in turn. Elastic tethering of the plate to an underlying foundation was shown to be effective in mediating pattern selection, with stiffening of the foundation promoting higher-order modes.

Localised tissue softening was assessed as one possible means for control of crypt distribution. Such a mechanism may be regarded as representative of the natural variations in matrix stiffness found in the developed crypt due to differential expression of growth factors such as TGF-β, whose abundant expression at the top of the crypt promotes collagen deposition, stiffening the matrix [55,56]. While localised softening exhibited some success in improving resemblance to colorectal crypts (Figs. 4(f) and 7(b)), it was shown that significant variations in the substrate’s stiffness were required to achieve this. For the parameters investigated, $R_{1}^{\left(1\right)}$ modes remained the lowest-energy configurations. The notion of attaining a regular array of crypts by prescribing a regular patterning of material softening alone seems infeasible, since the symmetries inherent in the model place restrictions upon the structure of permissible configurations. We note that a contributing factor here is our use of clamped boundary conditions, which are more appropriate to *in vitro* cell culture experiments than they are to the developing intestinal tissue *in vivo*. Under periodic boundary conditions, regular arrays of crypts are likely to be attained more easily.

Configurations attained under localised tissue softening were compared to those attained for a homogeneous substrate under the influence of spatially non-uniform cellular growth, accounting for strong adhesion between the cell layer and its substrate. Restricting cellular growth to discrete patches distributed over the layer showed some success in attaining the desired number of crypts. One-, four- and nine-crypt configurations were successfully determined in regimes with the corresponding number of growth patches (Fig. 6(a)–(c)). However, numerical simulations revealed that short-wavelength variations in the cells’ growth rate resulted in more complex configurations as buckled regions begin to interact (Fig. 6(d)). This model indicates that growth patterns can play a stronger role in pattern selection than suggested by the 1D models of [30]; in general, 1D models of this type fail to account fully for the complex energetic constraints associated with higher-dimensional models. Discrepancies in shape between *e.g.* Fig. 7 and fully-developed crypts *in vivo* can, in part, be attributed to our focus upon the onset of buckling. Future work will deploy this model in studying large-amplitude configurations, for which we anticipate inhomogeneous growth and tissue remodelling to play stronger roles in shape selection; it will also be important to investigate the role of secondary instabilities [9]. For reference, we provide in (B.8) a variational statement of the model, showing the additional contributions associated with the inter-layer coupling and forcing terms.

This model demonstrates the contributory effect of each pattern selection mechanism; however, for a full description of the *in vivo* system, it remains to couple this tissue-scale model’s inputs to biological phenomena at the cell scale. In particular, signalling between the various cell populations in the epithelium and underlying mucosa has been considered important; paneth cells, for example, are thought to provide signals that affect stem cell function [18,28]. A thorough understanding of the coupling between tissue-scale geometry, cell-scale regulations and biomechanical factors (*e.g.* growth rates, tissue stiffness) is currently unavailable, and is particularly difficult to elucidate at the developmental stage prior to crypt formation. Indeed, there is evidence to suggest that this coupling is truly bidirectional, tissue curvature being one input to paneth cell specification, and hence creation of the stem cell niche [28]. Developmentally, this suggests that the buckling phenomena described here are likely to occur initially as a precursor to more complex multiscale regulatory systems.

Investigation of the many complex interactions between tissue mechanics and intracellular signalling remains an active area of focus for both theoretical and experimental studies. In [11], for example, the roles of the Wnt and BMP signalling pathways in directing intestinal crypt formation are examined via a multiscale model that couples a reaction–diffusion description of the signalling proteins to a phenomenological description of the tissue mechanics. The model demonstrates the role of Wnt in determining the distribution of progenitor and differentiated cells, and the role of BMP in stabilising the crypt structure and preventing crypt fission. Furthermore, under periodic boundary conditions, the model shows that localised populations of stem cells can each give rise to a single crypt. This conclusion is consistent with the results of Fig. 6.

The results presented here are consistent with the hypothesis that intestinal crypts can form spontaneously in culture, given a sufficiently deformable substrate. However, while it seems that growing intestinal tissue *in vitro* may not require that the full three-dimensional architecture be imposed, it seems that the task of regenerating the distribution of crypts found *in vivo* may require a less simplistic culture platform than that described in [30]. In particular, we postulate that a flexible substrate supported by a fine degradable mesh that imposes periodicity upon buckled configurations may improve similarity to the *in vivo* tissue. Within such a culture system, Fig. 6 highlights strategic seeding of stem cells as a promising route to driving deformation in the required locations. The task of designing such scaffolds and protocols remains an area of focus for our experimental collaborators.

In summary, we have examined the independent effects of patterned growth and material properties in determining buckled shapes corresponding to multiple colorectal crypts. Unlike previous models that have focused upon patterning growth through chemical cues [11], or upon nonlinear feedback between growth and tissue configuration [29], we have assessed the relative strength of these mechanisms at the onset of buckling. Our simulations suggest that, in two dimensions, patterning of cellular growth constitutes a more viable mechanism for shape-selection than does material inhomogeneity.

## Figures and Tables

**Fig. 8:**
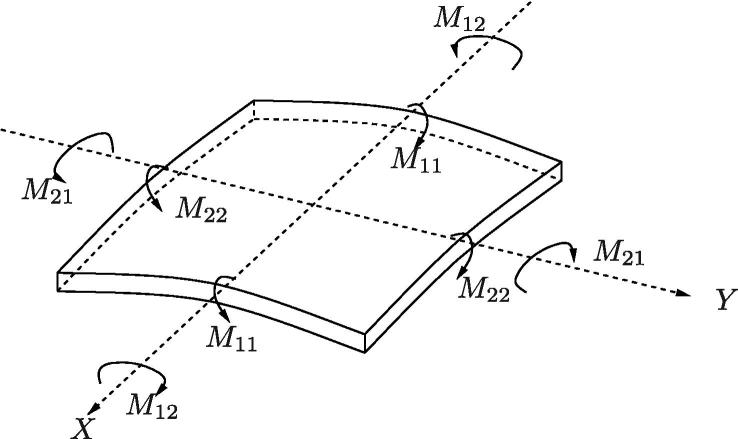
Moments acting upon a surface element.

**Table 1:** The first seven eigenvalues, *ξ*, of (5) and (7) with η=0 for each symmetry family. The numbers in square brackets denote the ordering of the eigenvalues, [1] being the eigenvalue of smallest magnitude. Stars denote eigenvalues with multiplicity 2 – the corresponding eigenmodes are identical, subject to a rotation through π/2 radians.

$R_{1}$	$R_{2}$	$R_{3}$	$R_{4}$	$R_{5}$
[1] 13.0862	[5] 38.5314	[12] 67.2802	[4] 32.0524	[2∗] 23.0311
[6] 41.7573	[16] 87.329	[25] 125.2549	[13] 69.7698	[7∗] 47.393
[11] 61.5818	[21] 106.3548	[32] 155.4244	[20] 100.9667	[9∗] 61.5806
[17] 90.6878	[33] 156.164	[46] 203.5528	[28] 127.9746	[14∗] 81.6609
[22] 108.6967	[38] 173.6856	[53] 232.2052	[34] 157.611	[18∗] 95.0768
[31] 150.212	[49] 214.4493	[68] 283.3951	[47] 206.1533	[23∗] 120.422
[35] 159.672	[57] 244.8421	[73] 301.8406	[48] 209.7246	[26∗] 125.9873

## Appendix A Derivation of the plate equations

We consider a thin, square plate of width $2L^{*}$ and height $h^{*}$. We denote the Lagrangian position of a material point by $\left(X_{1}^{*}\text{,}X_{2}^{*}\text{,}X_{3}^{*}\right)$, relative to fixed Cartesian coordinates oriented such that $X_{1}^{*}$ and $X_{2}^{*}$ lie in the plane of the undeformed plate, and $X_{3}^{*}$ is normal to the undeformed plate in a right-handed sense. The plate’s central plane lies at $X_{3}^{*}=0$ in the undeformed configuration. Similarly, we denote the Eulerian position of the same material point by $\left(x_{1}^{*}\text{,}x_{2}^{*}\text{,}x_{3}^{*}\right)$. The plate undergoes a deformation with Cartesian components of displacement $\left(u_{1}^{*}\text{,}u_{2}^{*}\text{,}u_{3}^{*}\right)$, so that

(A.1) $$x_{i}^{*}=X_{i}^{*}+u_{i}^{*}\text{.}$$

We describe stresses in terms of the first and second Piola–Kirchhoff stress tensors, denoted $T^{*}$ and $S^{*}$ respectively and related by

(A.2) $$S^{*}=F^{-1}T^{*}\text{,}$$

for deformation gradient tensor F. In general $T^{*}$ is not symmetric; however, (A.2) implies that its components are related via

(A.3) $$T^{*}F^{⊤}={\mathrm{FT}}^{*⊤}\text{.}$$

We denote the Lagrangian strain tensor by e, given by

(A.4) $$e=\frac{1}{2}\left(F^{⊤}F-I\right)\text{.}$$

Restricting attention to deflections of $O(h^{*})$, for which strains are small, we adopt a neo-Hookean stress–strain relationship given by

(A.5) $$S^{*}=\lambda^{*}\mathrm{Tr}(e)I+2\mu^{*}e\text{,}$$

where the Lamé moduli $\lambda^{*}$ and $\mu^{*}$ are given by

(A.6) $$\lambda^{*}=\frac{\nu E^{*}}{\left(1+\nu )(1-2\nu \right)}\text{,}\;\mu^{*}=\frac{E^{*}}{2\left(1+\nu \right)}\text{,}$$

provided ν≠0.5. It can be shown that in the incompressible limit (ν→0.5), Tr(e)→0 so that the stresses prescribed by (A.5) are bounded [59]. The equations derived below are, therefore, regular in this limit.

We select boundary conditions which impose clamping and zero displacement upon all four of the plate’s boundaries, *i.e.*

(A.7a) $$u_{i}^{*}\left(\pm L^{*}\text{,}X_{2}^{*}\text{,}X_{3}^{*}\right)=u_{i}^{*}\left(X_{1}^{*}\text{,}\pm L^{*}\text{,}X_{3}^{*}\right)=0\;\mathrm{for}\;i=1\text{,}2\text{,}3\text{,}$$

(A.7b) $$\frac{\partial u_{3}^{*}}{\partial X_{1}^{*}}\left(\pm L^{*}\text{,}X_{2}^{*}\text{,}X_{3}^{*}\right)=\frac{\partial u_{3}^{*}}{\partial X_{2}^{*}}\left(X_{1}^{*}\text{,}\pm L^{*}\text{,}X_{3}^{*}\right)=0\text{.}$$

We denote stresses applied at the plate’s upper and lower surfaces by $f^{+*}$ and $f^{-*}$ respectively. The remaining boundary conditions are then given by

(A.8) $$T_{i3}^{*}\left(X_{1}^{*}\text{,}X_{2}^{*}\text{,}\pm h^{*}/2\right)=\pm f_{i}^{\pm *}\left(X_{1}^{*}\text{,}X_{2}^{*}\right)\;\mathrm{for}\;i=1\text{,}2\text{,}3\text{,}$$

### A.1 Nondimensionalisation and asymptotic scalings

We nondimensionalise the system via the following scalings, which depend upon the slenderness parameter $\varepsilon =h^{*}/L^{*}$. We scale coordinates according to

(A.9) $$X_{1}^{*}=L^{*}X\text{,}\;X_{2}^{*}=L^{*}Y\text{,}\;X_{3}^{*}=h^{*}Z\text{,}$$

so that the plate is bounded by -1⩽X,Y⩽1 and -1/2⩽Z≤1/2. Following [42], we assume that deformations in the *Z*-direction are of small amplitude. Transverse displacements are assumed to be of $O(h^{*})$ and the associated in-plane displacements are assumed to be of $O(\varepsilon h^{*})$. We also retain an $O(\varepsilon^{2})$ correction to the leading-order transverse displacement, setting

(A.10) $$u_{1}^{*}=\varepsilon h^{*}u\text{,}\;u_{2}^{*}=\varepsilon h^{*}v\text{,}\;u_{3}^{*}=h^{*}\left(w+\varepsilon^{2}\overset{˜}{w}\right)\text{.}$$

We regard *w* to be independent of *Z*, which is equivalent to the assumption that the thickness of the plate is unchanged to leading-order during deformation. For compactness of notation, we abbreviate some of the equations below by denoting derivatives of displacements by appropriate subscripts. Noting (A.9) and (A.10), we determine the form of the deformation gradient tensor as

(A.11) $$F=\left(\begin{array}{ccc} 1+\varepsilon^{2}u_{X} & \varepsilon^{2}u_{Y} & \varepsilon u_{Z}\\ \varepsilon^{2}v_{X} & 1+\varepsilon^{2}v_{Y} & \varepsilon v_{Z}\\ \varepsilon w_{X}+\varepsilon^{3}{\overset{˜}{w}}_{X} & \varepsilon w_{Y}+\varepsilon^{3}{\overset{˜}{w}}_{Y} & 1+\varepsilon^{2}{\overset{˜}{w}}_{Z} \end{array}\right)\text{,}$$

and from (A.4) we have the following expression for the leading order Lagrangian strain:

(A.12) $$e=\frac{\varepsilon^{2}}{2}\left(\begin{array}{ccc} 2u_{X}+w_{X}^{2} & u_{Y}+v_{X}+w_{X}w_{Y} & 0\\ u_{Y}+v_{X}+w_{X}w_{Y} & 2v_{Y}+w_{Y}^{2} & 0\\ 0 & 0 & 2{\overset{˜}{w}}_{Z}+u_{Z}^{2}+v_{Z}^{2} \end{array}\right)\text{.}$$

We scale the Young’s modulus against its maximum, $E_{\max}^{*}$, setting

(A.13) $$E^{*}=E_{\max}^{*}E\text{.}$$

We adopt the scalings of the stress tensor components given by Howell et al. [42], which are

(A.14a) $$\left\{T_{\mathrm{ij}}^{*}\text{,}S_{\mathrm{ij}}^{*}\right\}=\varepsilon^{2}E_{\max}^{*}\left\{T_{\mathrm{ij}}\text{,}S_{\mathrm{ij}}\right\}\text{,}$$

(A.14b) $$\left\{T_{i3}^{*}\text{,}T_{3i}^{*}\text{,}S_{i3}^{*}\text{,}S_{3i}^{*}\right\}=\varepsilon^{3}E_{\max}^{*}\left\{T_{i3}\text{,}T_{3i}\text{,}S_{i3}\text{,}S_{3i}\right\}\text{,}$$

(A.14c) $$\left\{T_{33}^{*}\text{,}S_{33}^{*}\right\}=\varepsilon^{4}E_{\max}^{*}\left\{T_{33}\text{,}S_{33}\right\}\text{,}$$

for i,j=1,2. The corresponding rescalings of the components of $f^{\pm *}$ are

(A.15) $$f_{1}^{\pm *}=\varepsilon^{3}E_{\max}^{*}f_{1}^{\pm }\text{,}\;f_{2}^{\pm *}=\varepsilon^{3}E_{\max}^{*}f_{2}^{\pm }\text{,}\;f_{3}^{\pm *}=\varepsilon^{4}E_{\max}^{*}f_{3}^{\pm }\text{.}$$

Howell et al. [42] showed that for (A.14) to be consistent with (A.2) we require the following conditions upon the displacements:

(A.16a) $$u=\overset{¯}{u}(X\text{,}Y)-{\mathrm{Zw}}_{X}\text{,}\;v=\overset{¯}{v}(X\text{,}Y)-{\mathrm{Zw}}_{Y}\text{,}$$

(A.16b) $${\overset{˜}{w}}_{Z}=-\frac{w_{X}^{2}+w_{Y}^{2}+2\nu \left(u_{X}+v_{Y}\right)}{2(1-\nu )}\text{.}$$

Eq. (A.16) enforce the *Kirchhoff assumptions*, which state that (i) normals to the central plane in the undeformed configuration remain normal and inextensional throughout deformation, which follows from (A.16a), and (ii) transverse normal stresses are negligibly small in comparison with in-plane normal stress components, which follows from (A.16b).

Dimensionless boundary conditions are given by

(A.17a) u(±1,Y,Z)=u(X,±1,Z)=0,

(A.17b) v(±1,Y,Z)=v(X,±1,Z)=0,

(A.17c) w(±1,Y,Z)=w(X,±1,Z)=0,

(A.17d) $$w_{X}(\pm 1\text{,}Y\text{,}Z)=w_{Y}(X\text{,}\pm 1\text{,}Z)=0\text{,}$$

(A.17e) $$T_{i3}(X\text{,}Y\text{,}\pm 1/2)=\pm f_{i}^{\pm }(X\text{,}Y)\;\mathrm{for}\;i=1\text{,}2\text{,}3\text{.}$$

### A.2 Model derivation

In the absence of body forces, the above scalings reduce Cauchy’s momentum equations to

(A.18) $$\frac{\partial T_{i1}}{\partial X}+\frac{\partial T_{i2}}{\partial Y}+\frac{\partial T_{i3}}{\partial Z}=0\text{,}\;i=1\text{,}2\text{,}3\text{.}$$

We integrate (A.18) with respect to *Z* to obtain expressions for the averaged stress components, denoted with overbars and defined according to

(A.19) $${\overset{¯}{T}}_{\mathrm{ij}}=\int_{-1/2}^{1/2}T_{\mathrm{ij}}dZ\text{.}$$

Since the boundary conditions given by (A.17e) imply that

(A.20) $$\frac{\partial {\overset{¯}{T}}_{i3}}{\partial Z}=\int_{-1/2}^{1/2}\frac{\partial T_{i3}}{\partial Z}dZ={\left[T_{i3}\right]}_{-1/2}^{1/2}=f_{i}^{+}+f_{i}^{-}\text{,}\;i=1\text{,}2\text{,}3\text{,}$$

the expressions resulting from (A.18) are as follows:

(A.21) $$\frac{\partial {\overset{¯}{T}}_{i1}}{\partial X}+\frac{\partial {\overset{¯}{T}}_{i2}}{\partial Y}+f_{i}^{+}+f_{i}^{-}=0\text{,}\;i=1\text{,}2\text{,}3\text{.}$$

Similarly, multiplying the first two expressions in (A.18) by *Z* and integrating yields equations for the bending moments,

(A.22) $$\frac{\partial M_{i1}}{\partial X}+\frac{\partial M_{i2}}{\partial Y}-{\overset{¯}{T}}_{i3}+\frac{1}{2}\left(f_{i}^{+}-f_{i}^{-}\right)=0\text{,}\;i=1\text{,}2\text{,}$$

where, using the notation of [60],

(A.23) $$M_{\mathrm{ij}}=\int_{-1/2}^{1/2}T_{\mathrm{ij}}ZdZ\text{,}$$

the senses of which are illustrated in Fig. 8.

We can eliminate ${\overset{¯}{T}}_{21}\text{,}{\overset{¯}{T}}_{31}$ and ${\overset{¯}{T}}_{32}$ from the system since (A.3) and (A.11) imply that

(A.24a) $${\overset{¯}{T}}_{21}={\overset{¯}{T}}_{12}+O\left(\varepsilon^{2}\right)\text{,}$$

(A.24b) $${\overset{¯}{T}}_{31}={\overset{¯}{T}}_{13}+w_{X}{\overset{¯}{T}}_{11}+w_{Y}{\overset{¯}{T}}_{12}+O\left(\varepsilon^{2}\right)\text{,}$$

(A.24c) $${\overset{¯}{T}}_{32}={\overset{¯}{T}}_{23}+w_{X}{\overset{¯}{T}}_{21}+w_{Y}{\overset{¯}{T}}_{22}+O\left(\varepsilon^{2}\right)\text{.}$$

Eq. (A.24a) states that averaged in-plane shear stresses are equal to leading order.

For notational convenience we define a vector F with components given by

(A.25) $$F_{i}=f_{i}^{+}+f_{i}^{-}\;\mathrm{for}\;i=1\text{,}2\text{,}$$

representing the net in-plane stresses acting in each coordinate direction due to forcing on both surfaces of the plate. Deploying the Helmholtz decomposition theorem, which states that any two-dimensional vector field can be written as the sum of a conservative component and a rotational component [61], we write

(A.26) $$F=\nabla \chi +\nabla \times \left(\psi \overset{ˆ}{z}\right)\text{,}$$

for scalar fields *χ* and *ψ*. Under this definition, the first two expressions of (A.21) become

(A.27a) $$\frac{\partial }{\partial X}\left({\overset{¯}{T}}_{11}+\chi \right)+\frac{\partial }{\partial Y}\left({\overset{¯}{T}}_{12}+\psi \right)=0\text{,}$$

(A.27b) $$\frac{\partial }{\partial Y}\left({\overset{¯}{T}}_{22}+\chi \right)+\frac{\partial }{\partial X}\left({\overset{¯}{T}}_{12}-\psi \right)=0\text{.}$$

We simplify (A.27) by writing the stress resultants in terms of some Airy stress function Φ(X,Y). However, (A.27) suggests that the definition of a self-consistent Airy stress function is not possible for non-zero ψ. We, thus, restrict our attention to the case ψ≡0. From (A.27) we deduce the appropriate definition of Φ(X,Y) to be

(A.28) $${\overset{¯}{T}}_{11}=\frac{\partial^{2}\Phi }{\partial Y^{2}}-\chi (X\text{,}Y)\text{,}\;{\overset{¯}{T}}_{22}=\frac{\partial^{2}\Phi }{\partial X^{2}}-\chi (X\text{,}Y)\text{,}\;{\overset{¯}{T}}_{12}=-\frac{\partial^{2}\Phi }{\partial X\partial Y}\text{.}$$

Due to (A.28), the first two expressions of (A.21) are satisfied trivially. For convenience, we define the vector **Ω** to represent the couple induced by in-plane surface stresses, *i.e*

(A.29) $$\Omega_{i}=f_{i}^{+}-f_{i}^{-}\;\mathrm{for}\;i=1\text{,}2\text{.}$$

Also, we denote by N the total normal force applied to the upper and lower surfaces of the plate, *i.e*

(A.30) $$N=f_{3}^{+}+f_{3}^{-}\text{.}$$

Using (A.22), (A.23), (A.24a)–(A.24c), (A.25), (A.26), (A.27a), (A.27b), (A.28)–(A.30) to manipulate the final expression of (A.21), we obtain

(A.31) $$\begin{array}{c} \frac{\partial^{2}M_{11}}{\partial X^{2}}+2\frac{\partial^{2}M_{12}}{\partial X\partial Y}+\frac{\partial^{2}M_{22}}{\partial Y^{2}}+\frac{\partial^{2}w}{\partial X^{2}}\frac{\partial^{2}\Phi }{\partial Y^{2}}-2\frac{\partial^{2}w}{\partial X\partial Y}\frac{\partial^{2}\Phi }{\partial X\partial Y}+\frac{\partial^{2}w}{\partial Y^{2}}\frac{\partial^{2}\Phi }{\partial X^{2}}\\ +\frac{1}{2}\nabla \cdot \Omega -\nabla \cdot \left(\chi \nabla w\right)+N=0\text{.} \end{array}$$

Utilising (A.16), we calculate the remaining stress components as

(A.32a) $$T_{11}=E\left(\frac{2u_{X}+w_{X}^{2}+\nu \left(2v_{Y}+w_{Y}^{2}\right)}{2\left(1-\nu^{2}\right)}\right)+O\left(\varepsilon^{2}\right)\text{,}$$

(A.32b) $$T_{12}=E\left(\frac{u_{Y}+v_{X}+w_{X}w_{Y}}{2\left(1+\nu \right)}\right)+O\left(\varepsilon^{2}\right)\text{,}$$

(A.32c) $$T_{22}=E\left(\frac{\nu \left(2u_{X}+w_{X}^{2}\right)+2v_{Y}+w_{Y}^{2}}{2\left(1-\nu^{2}\right)}\right)+O\left(\varepsilon^{2}\right)\text{.}$$

Averaging (A.32), we obtain

(A.33a) $${\overset{¯}{T}}_{11}\equiv \frac{\partial^{2}\Phi }{\partial Y^{2}}-\chi =E\left(\frac{2{\overset{¯}{u}}_{X}+w_{X}^{2}+\nu \left(2{\overset{¯}{v}}_{Y}+w_{Y}^{2}\right)}{2\left(1-\nu^{2}\right)}\right)\text{,}$$

(A.33b) $${\overset{¯}{T}}_{12}\equiv -\frac{\partial^{2}\Phi }{\partial X\partial Y}=E\left(\frac{{\overset{¯}{u}}_{Y}+{\overset{¯}{v}}_{X}+w_{X}w_{Y}}{2\left(1+\nu \right)}\right)\text{,}$$

(A.33c) $${\overset{¯}{T}}_{22}\equiv \frac{\partial^{2}\Phi }{\partial X^{2}}-\chi =E\left(\frac{\nu \left(2{\overset{¯}{u}}_{X}+w_{X}^{2}\right)+2{\overset{¯}{v}}_{Y}+w_{Y}^{2}}{2\left(1-\nu^{2}\right)}\right)\text{.}$$

Rearranging (A.33a) and (A.33c) gives that

(A.34a) $${\overset{¯}{u}}_{X}+\frac{w_{X}^{2}}{2}=\frac{1}{E}\left(\frac{\partial^{2}\Phi }{\partial^{2}Y}-\nu \frac{\partial^{2}\Phi }{\partial^{2}X}-\left(1-\nu \right)\chi \right)\text{,}$$

(A.34b) $${\overset{¯}{v}}_{Y}+\frac{w_{Y}^{2}}{2}=\frac{1}{E}\left(\frac{\partial^{2}\Phi }{\partial^{2}X}-\nu \frac{\partial^{2}\Phi }{\partial^{2}Y}-\left(1-\nu \right)\chi \right)\text{.}$$

We eliminate $\overset{¯}{u}$ and $\overset{¯}{v}$ from the system by differentiating (A.33b) with respect to *X* and *Y* and noting (A.34), giving (3a) and (4).

The bending moments are evaluated by multiplying (A.32) by *Z* and integrating with respect to *Z*, yielding

(A.35a) $$M_{11}=-\frac{E}{12\left(1-\nu^{2}\right)}\left(\frac{\partial^{2}w}{\partial X^{2}}+\nu \frac{\partial^{2}w}{\partial Y^{2}}\right)\text{,}$$

(A.35b) $$M_{12}=-\frac{E}{12\left(1+\nu \right)}\frac{\partial^{2}w}{\partial X\partial Y}\text{,}$$

(A.35c) $$M_{22}=-\frac{E}{12\left(1-\nu^{2}\right)}\left(\frac{\partial^{2}w}{\partial Y^{2}}+\nu \frac{\partial^{2}w}{\partial X^{2}}\right)\text{.}$$

Substituting (A.35) into (A.31), we obtain (3b).

## Appendix B Incorporating growth

We may extend the model derived in Appendix A.2 to incorporate growth, allowing the resulting equations to be used to describe a proliferating cell monolayer, as well as the substrate upon which it rests.

Following the formalism of Skalak & Rodriguez [35–37], we consider a multiplicative decomposition of the deformation gradient tensor of the form F=AG, where G represents volumetric growth and A describes resulting elastic deformations that correct for any growth-induced incompatibilities. For small deflections, we write A=I+δa and G=I+δg for some 0<δ≪1. Eq. (A.4) then admits the following form of the strain tensor e to leading order:

(B.1) $$e=\frac{\delta }{2}\left(a^{⊤}+a+g^{⊤}+g\right)\equiv e_{0}-e_{G}\text{,}$$

where $e_{0}=\delta (a^{⊤}+a)/2$ accounts for strain due to elastic deformations, and is of the form given in (A.12), and $e_{G}=-\delta (g^{⊤}+g)/2$ accounts for strain induced by growth. Substituting (B.1) into (A.5) then motivates the following stress–strain relationship

(B.2) $$S^{*}=\lambda^{*}\mathrm{Tr}(e_{0})I+2\mu^{*}e_{0}-G^{*}\text{.}$$

where $G^{*}=\lambda^{*}\mathrm{Tr}\left(e_{G}\right)+2\mu^{*}e_{G}$.

Eq. (B.2) recovers the stress–strain relationship used in the linearly elastic model of [62], in which $G^{*}$ is considered to be of diagonal form. However, [63] suggests that for a plate under clamped boundary conditions undergoing deformations in the weakly nonlinear regime, one does not expect $G^{*}$ to be diagonal in general. We incorporate a fully non-zero $G^{*}$ here for generality, but restrict attention to the case in which the growth tensor has no *Z*-dependence and creates no changes in the thickness of the plate. Thus, we apply the following scalings:

(B.3a) $$\left\{G_{11}^{*}\text{,}G_{12}^{*}\text{,}G_{21}^{*}\text{,}G_{22}^{*}\right\}=E_{\max}^{*}\varepsilon^{2}\left\{G_{11}\text{,}G_{12}\text{,}G_{21}\text{,}G_{22}\right\}\text{,}$$

(B.3b) $$\left\{G_{13}^{*}\text{,}G_{23}^{*}\text{,}G_{31}^{*}\text{,}G_{32}^{*}\right\}=E_{\max}^{*}\varepsilon^{3}\left\{G_{13}\text{,}G_{23}\text{,}G_{31}\text{,}G_{32}\right\}\text{,}$$

(B.3c) $$G_{33}^{*}=E_{\max}^{*}\varepsilon^{4}G_{33}\text{,}$$

which preserve the balance of terms given in (A.14) and (A.16).

Repeating the derivation of Appendix A.2 with the additional growth term included, (A.33) becomes

(B.4a) $${\overset{¯}{T}}_{11}\equiv \frac{\partial^{2}\Phi }{\partial Y^{2}}-\chi =E\left(\frac{2{\overset{¯}{u}}_{X}+w_{X}^{2}+\nu \left(2{\overset{¯}{v}}_{Y}+w_{Y}^{2}\right)}{2\left(1-\nu^{2}\right)}\right)-G_{11}\text{,}$$

(B.4b) $${\overset{¯}{T}}_{12}\equiv -\frac{\partial^{2}\Phi }{\partial X\partial Y}=E\left(\frac{{\overset{¯}{u}}_{Y}+{\overset{¯}{v}}_{X}+w_{X}w_{Y}}{2\left(1+\nu \right)}\right)-G_{12}\text{,}$$

(B.4c) $${\overset{¯}{T}}_{22}\equiv \frac{\partial^{2}\Phi }{\partial X^{2}}-\chi =E\left(\frac{\nu \left(2{\overset{¯}{u}}_{X}+w_{X}^{2}\right)+2{\overset{¯}{v}}_{Y}+w_{Y}^{2}}{2\left(1-\nu^{2}\right)}\right)-G_{22}\text{,}$$

which may be rearranged to obtain an expression equivalent to (A.34):

(B.5a) $${\overset{¯}{u}}_{X}+\frac{w_{X}^{2}}{2}=\frac{1}{E}\left(\frac{\partial^{2}\Phi }{\partial^{2}Y}-\nu \frac{\partial^{2}\Phi }{\partial^{2}X}-\left(1-\nu \right)\chi +G_{11}-\nu G_{22}\right)\text{,}$$

(B.5b) $${\overset{¯}{v}}_{Y}+\frac{w_{Y}^{2}}{2}=\frac{1}{E}\left(\frac{\partial^{2}\Phi }{\partial^{2}X}-\nu \frac{\partial^{2}\Phi }{\partial^{2}Y}-\left(1-\nu \right)\chi +G_{22}-\nu G_{11}\right)\text{.}$$

Using (B.5) to eliminate $\overset{¯}{u}$ and $\overset{¯}{v}$ from (B.4) gives the following equation for Φ, which extends (3a):

(B.6) $$\nabla^{2}\left(\frac{1}{E}\nabla^{2}\Phi \right)-\left[\frac{1+\nu }{E}\text{,}\Phi \right]-\nabla^{2}\left(\frac{1-\nu }{E}\chi \right)+\frac{1}{2}\left[w\text{,}w\right]+\frac{G}{E}=0\text{,}$$

where

(B.7) $$G=\frac{\partial^{2}}{\partial X^{2}}\left(G_{22}-\nu G_{11}\right)+\frac{\partial^{2}}{\partial Y^{2}}\left(G_{11}-\nu G_{22}\right)-\frac{\partial^{2}G_{12}}{\partial X\partial Y}\text{.}$$

Since growth is independent of *Z*, it does not generate bending moments, and (3b) is unchanged. We note that if growth is in-plane isotropic, then $G=\left(1-\nu \right)\nabla^{2}g$, for some scalar *g*.

Finally, we note that the system given by (3a) and (B.6) may also be derived via a variational approach based upon the following functional:

(B.8) $$\begin{array}{cc} I=\int_{-1}^{1}\int_{-1}^{1} & \frac{E}{24\left(1-\nu^{2}\right)}{\left(\nabla^{2}w\right)}^{2}-\frac{1}{2E}{\left(\nabla^{2}\Phi \right)}^{2}-\frac{1}{2}\Phi \left[w\text{,}w\right]\\ & +\frac{1}{2}\left(\frac{1+\nu }{E}\right)\left[\Phi \text{,}\Phi \right]-\frac{G}{E}\Phi -\left(\frac{E}{24\left(1+\nu \right)}\right)\left[w\text{,}w\right]\\ & +\nabla^{2}\left(\frac{1-\nu }{E}\chi \right)\Phi -\frac{1}{2}\left(\nabla \cdot \Omega \right)w-\frac{\chi }{2}\left(w_{X}^{2}+w_{Y}^{2}\right)-Nw\;dXdY\text{.} \end{array}$$

While attributing physical meaning to every term in (B.8) is difficult, we note in particular that the first three contributions represent bending, stretching and a penalty for inducing Gaussian curvature respectively.
